# Management Strategies for Older Patients with Low-Risk Early-Stage Breast Cancer: A Physician Survey

**DOI:** 10.3390/curroncol29010001

**Published:** 2021-12-21

**Authors:** Mashari Alzahrani, Mark Clemons, Lynn Chang, Lisa Vendermeer, Angel Arnaout, Gail Larocque, Katherine Cole, Tina Hsu, Deanna Saunders, Marie-France Savard

**Affiliations:** 1 Department of Medicine, Division of Medical Oncology, The Ottawa Hospital and The University of Ottawa, Ottawa, ON K1H 8L6, Canada; mashari_220@hotmail.com (M.A.); mclemons@toh.ca (M.C.); katcole@toh.ca (K.C.); thsu@toh.ca (T.H.); 2 Cancer Therapeutics Program, Ottawa Hospital Research Institute, Ottawa, ON K1Y 4E9, Canada; lvandermeer@ohri.ca (L.V.); anarnaout@toh.ca (A.A.); dsaunders@ohri.ca (D.S.); 3 Department of Radiology, Division of Radiation Oncology, The Ottawa Hospital Cancer Centre and The University of Ottawa, Ottawa, ON K1H 8L6, Canada; lychang@toh.ca; 4 Department of Surgery, Division of General Surgery, The Ottawa Hospital and The University of Ottawa, Ottawa, ON K1H 8L6, Canada; 5 The Ottawa Hospital, Ottawa, ON K1H 8L6, Canada; galarocque@toh.ca

**Keywords:** breast cancer, radiation therapy, endocrine therapy, older patients, elderly, adjuvant

## Abstract

When managing older patients with lower-risk hormone-receptor-positive (HR+), HER2 negative (HER2−) early-stage breast cancer (EBC), the harms and benefits of adjuvant therapies should be taken into consideration. A survey was conducted among Canadian oncologists on the definitions of “low risk” and “older”, practice patterns, and future trial designs. We contacted 254 physicians and 21% completed the survey (50/242). Most respondents (68%, 34/50) agreed with the definition of “low risk” HR+/HER2− EBC being node-negative and either: ≤3 cm and low histological grade, ≤2 cm and intermediate grade, or ≤1 cm and high grade. The most popular chronological and biological age definition for older patients was ≥70 (45%, 22/49; 45% 21/47). In patients ≥ 70 with low risk EBC, most radiation and medical oncologists would recommend post-lumpectomy radiotherapy (RT) and endocrine therapy (ET). Seventy-eight percent (38/49) felt that trials are needed to evaluate RT and ET’s role in patients ≥ 70. The favored design was ET alone, vs. RT plus ET (39%, 15/38). The preferred primary and secondary endpoints were disease-free survival and quality of life, respectively. Although oncologists recommended both RT and ET, there is interest in performing de-escalation trials in patients ≥ 70.

## 1. Introduction

Managing older patients with early-stage breast cancer (EBC) requires a careful evaluation of the potential risks and benefits of treatment, as well as the integration of competing risks of non-cancer-related mortality. Although current guidelines recommend adjuvant radiotherapy (RT) and endocrine therapy (ET) for all hormone-receptor-positive (HR+) EBC [1], there may be some older patients, such as those with comorbid medical conditions, where the potential harms of RT and/or ET may outweigh the benefits [2,3,4].

A few trials have been performed to identify older patients with a lower risk EBC, for whom it might be appropriate to de-escalate adjuvant therapies. These trials tend to focus mainly on omitting RT [5,6,7]. Indeed, a recent systematic review that included seven randomized controlled trials (RCT) evaluated the omission of RT [8,9,10,11,12]. This review showed that in patients treated with adjuvant ET, the addition of adjuvant RT reduced in-breast tumor recurrence (IBTR) at 5 years (summary risk ratio (SRR) 0.15, 95% CI 0.08–0.28), but had no effect on survival (SRR 0.97, 95% CI 0.79–1.2) [13]. Only one RCT compared adjuvant RT alone, to either adjuvant ET alone, or to the standard of care (i.e., RT plus ET), and showed that RT alone was superior in preventing IBTR, compared with ET alone [8]. Considering the body of evidence, more trials on the omission of ET are required to better inform treatment decisions. Indeed, a recent study evaluating the benefits of adjuvant ET for EBC has shown that when competing risks for death are taken into account, not only are disease-free survival (DFS) and distant recurrence-free survival (DRFS) events significantly reduced, but the overall benefits of ET become more modest, particularly for older patients and those with lower-risk disease [14].

Despite the fact that over 30% of EBC are diagnosed in patients ≥ 70 years, there is still controversy around what defines the terms “older” and “low risk” HR+/HER2− breast cancer, and how to properly counsel these older patients on the optimal adjuvant strategy (i.e., RT alone, ET alone or RT + ET) [15,16,17]. We therefore surveyed oncologists regarding their definitions of the terms “low risk” and “older”, their current practice patterns, and their thoughts on potential future trial designs for older patients with low-risk node-negative, HR+/HER2− EBC. The information obtained from these surveys could assist in the design of future pragmatic clinical trials to evaluate the risks and benefits of adjuvant RT and ET in older patients with lower-risk breast cancer, with findings that are clinically relevant, patient-centered and practice-changing.

## 2. Materials and Methods

### 2.1. Survey Development

The survey was designed by a multidisciplinary team with established expertise in survey development and performance [18,19,20]. The target population was Canadian oncology clinicians (i.e., medical oncologists, radiation oncologists, surgical oncologists and general practitioners in oncology/radiation) involved in the treatment of patients with HR+/HER2− lymph-node-negative EBC. The first section of the survey collected pertinent respondent demographic information (5 items). The second section collected information on how clinicians defined the terms “low risk” and “older” (19 items). This included querying the definition of low-clinical-risk disease, proposed by Sparano et al. [17] which defined node-negative low-risk breast cancer as: a tumor ≤ 3 cm with a low histological grade, a tumor ≤ 2 cm with intermediate grade, or a tumor ≤ 1 cm with high grade. In the third section, medical oncologist and radiation oncologists were asked about their personal practice regarding the use of RT and ET in older patients (15 items). Finally, respondents were presented with different scenarios for a potential clinical trial in older patients with node-negative HR+/HER2− low-risk breast cancer. They were also asked for their opinion on an acceptable primary study endpoint that could inform a change in their practice (16 items). The survey was pilot tested on three oncologists, a nurse practitioner and two research staff before launch. The survey is shown in the Supplementary Materials.

### 2.2. Survey Implementation

Canadian physicians treating breast cancer were identified through publicly available physician email addresses, which were used in previous surveys of this type [18,19,20]. The online survey was run using Microsoft Forms from the designated research coordinator’s secure account, within the Ottawa Hospital Research Institute. The survey was initiated on 16 September 2020 and remained open until 23 November 2020. Physicians were sent an invitation to complete the survey, and a link to the electronic survey as well as an information sheet for the study. Completion of the survey implied consent to participate. A reminder notice was sent to participants 4 weeks later. The survey was approved by the Ontario Cancer Research Ethics Board (OCREB).

### 2.3. Data Analysis

All of the data was summarized descriptively. The frequency of each answer choice was tabulated as a proportion of the total number of respondents for that category. Data were analyzed using SPSS version 27 IBM (Armonk, NY, USA).

## 3. Results

### 3.1. Physician Demographics

The electronic survey was sent to 254 physicians; 12 invitees were not eligible or reachable (e.g., on maternity leave, no longer treating breast cancer, retired, or e-mail address invalid). Out of the 242 eligible physicians, 50 responded (21%), all of whom were involved in adjuvant ET/RT discussion with patients. The majority of eligible respondents (*n* = 50), 60% (30/49) were medical oncologists, 24% (12/49) radiation oncologists, 8% (4/49) surgical oncologists that initiate ET, and 6% (3/49) were general/nurse practitioners, or general internists (Table 1). Of these, 76% (38/50) worked in academic (teaching) hospitals. Their length of time in independent practice varied, with 28% (14/50) working 5 years or less, 18% (9/50) 6–10 years, 24% (12/50) 11–20 years and 30% (15/50) more than 20 years (Table 1).

### 3.2. Definition of the Term “Low Risk” HR+/HER− EBC

When respondents were asked about their opinion regarding the definition of low-risk HR+/HER2− EBC, 68% (34/50) agreed with the definition of low-clinical-risk disease proposed by Sparano et al. [20] (Table 2). Among 14 respondents who did not agree with this definition or were unsure, some respondents agreed with a broader definition: two respondents suggested that a tumor ≤ 5 cm with low histologic grade could also be considered as low-clinical-risk disease; two respondents would consider a tumor ≤ 3 cm with intermediate histologic grade as low-clinical-risk disease and two respondents would consider a tumor ≤ 2 cm with high histologic grade as low-clinical-risk disease. Furthermore, seven respondents (7/50, 14%) would exclude patients with any grade tumor larger than 2 cm from the low-risk definition. When respondents were asked about multifocal EBC, 73% (36/49) suggested these EBC patients could still be considered low risk.

In regard to whether Ki-67 (protein expressed in the nucleus of cells during different phases of the cell cycle; principally used to evaluate prognosis) is required to define low-risk breast cancer, 56% (28/50) thought Ki-67 would improve their confidence in identifying low-risk breast cancer but that it was not absolutely necessary. When asked if one of the multigene profiling assays (MPA) such as Oncotype Dx Recurrence Score (RS), EndoPredict and Prosigna was required to define lower-risk breast cancer irrespective of tumor size and grade, 50% (25/50) indicated that MPA would improve their confidence in identifying low-risk breast cancer, but that it was not absolutely necessary, 20% (10/50) would not feel comfortable defining low risk without MPA, and 26% (13/50) did not think an MPA was necessary for identifying low-risk breast cancer. Regarding the preferred type of MPA, 74% (26/35) preferred using Oncotype Dx Recurrence Score (RS) to identify low-risk breast cancer.

### 3.3. Definition of the Term “Older”

When respondents were asked about what chronological age (i.e., age in terms of years since birth) they would define as “older”, 45% (22/49) indicated ≥ 70 years, 20% (10/49) indicated ≥ 75 years and 31% (15/49) indicated ≥ 80 years. When respondents were asked about what biological age (also termed “physiological age” and reflecting a patients physical and mental function) they would define as older, 45% (21/47) indicated ≥ 70 years, 15% (7/47) suggested ≥ 75 years, and 30% (14/47) suggested a biological age of ≥80. Table 2.

### 3.4. Practice Regarding the Use of RT in Older Patients

All the physicians were asked at what age they would consider de-escalating (i.e., partial breast radiation, altered fractionation) and/or omitting RT and the majority (37% (15/41)) mentioned 70 years old (chronological or biological age) (Table 3).

The most popular RT regimens in patients ≥ 70 years with low risk, node-negative ER+/HER2− EBC were: 5 days per week for 3 weeks with or without boost (25%, 3/12); accelerated partial breast radiation with external beam or brachytherapy (33%, 4/12); standard fractionation partial breast, also known as IMPORT LOW (8%, 1/12), and the FAST-FORWARD protocol (33%, 4/12). When asked if they would offer radiation boost to the above regimens, 83% (10/12) of respondents would decide based on pathology results (e.g., margins, grade, LVI) and 17% (2/12) would not offer radiation boost.

Issues with RT compliance among patients ≥ 70 years was reported to be <1% by 33% (4/12) of respondents, 1% to <5% by 42% (5/12), and more than 10% by 8% (1/12). Indeed, 17% (2/12) of respondents never encountered issues with compliance during their practice. In patients ≥ 70 years, RT tolerability is believed to be worse compared with younger patients for 25% (3/12) of respondents, whereas 75% (9/12) believed tolerability was not worse, or the same, as seen in Table 3.

### 3.5. Practice Regarding the Use of ET in Older Patients

In patients ≥ 70 years of age with low-risk, node-negative ER+/HER2− breast cancer, ET was generally recommended by 57% (25/44) of participants that ET be initiated. One fifth (20%; 9/44) strongly recommend ET. Fifty-seven percent (25/44) generally recommend ET, and 16% (7/44) did not offer ET if the patient had multiple co-morbidities. Seven percent (3/44) discussed the risks and benefits with the patient. Physicians would consider de-escalating (i.e., a shorter duration of therapy) and/or omitting ET at age 70 (16% (7/43)), 75 (14% (6/43)), 80 (30% (13/43)), and 85 (28% (12/43)) (chronological or biological age), respectively.

Regarding the type of ET respondents would use in this population, 49% (22/45), 31% (14/45) and 13% (6/45) would prescribe an aromatase inhibitor (AI), switch strategy (Tamoxifen followed by AI, or vice-versa) and tamoxifen, respectively. The majority of respondents (93% (41/44)) would recommend 5 years of ET. Of these, 63% (26/41) indicated a low threshold to stop therapy if side effects occurred (Table 3).

**Figure 1:**
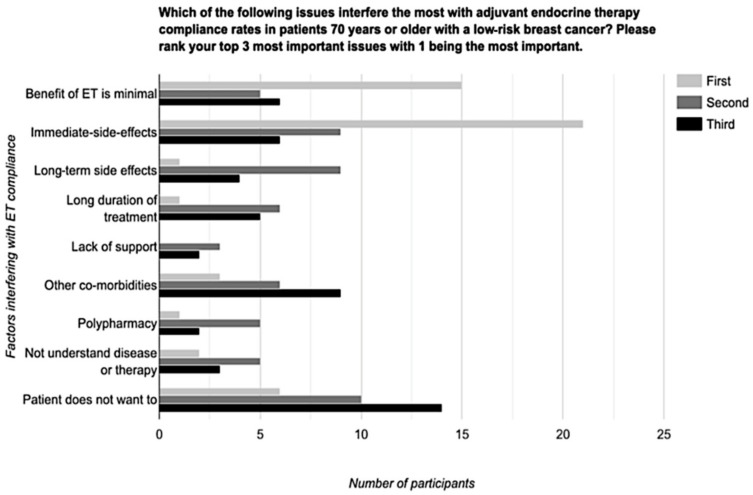
Factors that oncologists identified as interfering with ET compliance in patients aged ≥ 70 with a low-risk breast cancer.

With respect to compliance, in those older patients ≥ 70 years for whom ET was recommended, 43% (20/47) of respondents thought < 10% of patients ≥ 70 years did not actually ever start ET, while 47% (22/47) thought < 25% of patients would never start their ET. With respect to adherence, 41% (19/46) and 39% (18/46) suggested that ≥50% and ≥75% of patients were fully adherent to their ET, respectively (Table 3). When respondents were asked about how often they thought patients ≥ 70 years stopped taking their ET earlier than the originally prescribed duration, 14% (6/44) suggested < 10% of patients, 41% (18/44) suggested < 25% of patients, and 30% (13/44) suggested < 50% of patients stop ET early. The factors that interfere with ET compliance are shown in Figure 1. In patients ≥ 70 years, the majority of respondents (74%; 35/47) believed that ET tolerability was similar to younger patients, whereas 15% (7/47) felt that it was worse, and 11% (5/47) were unsure.

### 3.6. Impact of Axillary Surgery on Decision of Adjuvant RT and ET

For patients ≥ 70 years that were not receiving sentinel node biopsy or any form of axillary surgery, as per the Society of Surgical Oncology Guidelines [21], 45% (20/44) of respondents were more likely to give ET, whereas 55% (24/44) indicated that this did not affect their decision. With regards to RT, 50% (6/12) of respondents were more likely to give RT if surgical evaluation of axilla was not offered, whereas 42% (5/12) indicated that this did not affect their decision.

### 3.7. Potential Future De-Escalation Trials of Adjuvant RT and/or ET in Older Patients with Low-Risk ER+/HER2− EBC

Respondents were asked for their views on potential future de-escalation trials of adjuvant ET and/or RT, and the relevance of various study endpoints to facilitate the design of practice-changing trials. Three quarters (78% (38/49)]) of respondents felt trials to evaluate the risks and benefits of RT and ET in older patients with low-risk ER+/HER2− EBC are needed, whereas 12% (6/49) felt such trials are not needed, and 10% (5/49) were unsure (Table 4).

**Figure 2:**
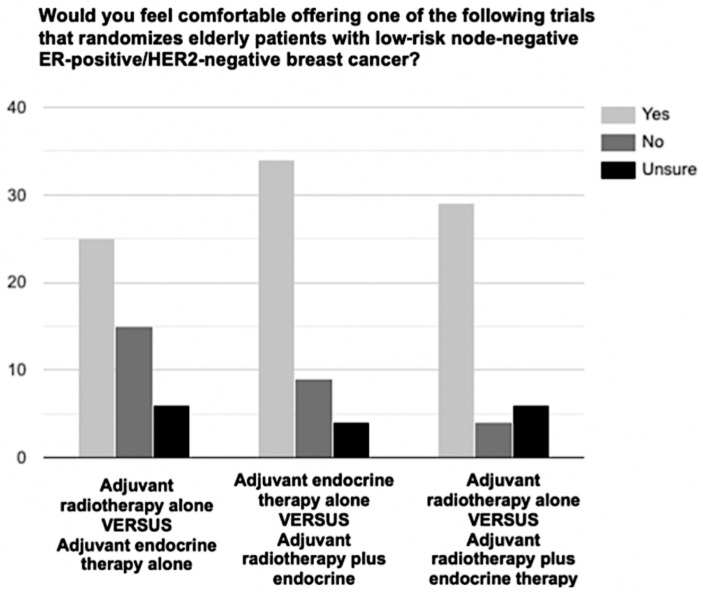
Oncologists comfort in offering future de-escalation trials designs.

Respondents were offered three trial designs, to choose the most informative and impactful design based on their knowledge, experience and current practice. Thirty-nine percent (15/38) suggested adjuvant ET alone vs. adjuvant RT plus ET, 32% (12/38) suggested adjuvant RT alone vs. adjuvant RT plus ET, and 29% (11/38) suggested adjuvant RT alone vs. adjuvant ET alone (Table 4). When respondents were asked if they felt comfortable offering one of the above trials to their older patients, 72% (34/47), 64% (29/45) and 54% (25/46) said they would feel comfortable offering the previously mentioned trials to their patients, respectively (Figure 2).

Respondents were asked for their opinion of the most appropriate primary endpoint in a study evaluating risks and benefits of ET in older patients with low-risk ER+/HER2− EBC. The most commonly chosen endpoint was DFS (25% (12/48)), followed by overall survival (OS) (19% (9/48)), breast cancer specific survival (17% (8/48)), and DRFS (15% (7/48)) (Table 4). The most important secondary endpoints chosen were indicators of health quality of life, using the EORTC-QLQ-C30 questionnaire (54%, 26/48) and OS (41%, 20/48) (Table 4).

## 4. Discussion

Developing evidence-based guidelines for the treatment of older patients with breast cancer can be challenging for a number of reasons. First is the broad spectrum of functional status and other health issues in this aging population. Second, older patients are often underrepresented in studies, as they have been excluded from many practice-changing clinical trials because of their age or co-morbidities [16]. Finally, some studies suggest the existence of a less aggressive breast cancer biology in most patients ≥ 70 years of age [14]. Therefore, it is not surprising that evidence-based guidelines regarding the optimal treatment of older patients with EBC are lacking [22,23,24].

This survey was devised to gain an understanding of current Canadian practice patterns in older patients with low-risk, ER+/HER2− EBC. Although the definition of low-risk HR+ HER2− EBC varies in studies, a majority of respondents agreed with the definition proposed by Sparano et al. [17], which defined low-risk breast cancer as: a tumor ≤ 3 cm with a low histological grade, a tumor ≤ 2 cm with an intermediate grade, or a tumor ≤ 1 cm with a high grade. The majority of respondents indicated the age of 70 as both the chronological and biological age for defining “older adults”, with chronological age referring to the amount of time a person has lived, and biological age relating to the presence of diseases associated with old age and the decline of function [25]. In clinic, the concept of biological age is often used loosely. Recently, epigenetic changes and DNA-methylation has been used to calculate biological age, but its use remains limited in gerontology research and is not broadly applied in the clinical setting [26]. Therefore, as reflected by the wide range of answers, there is a lack of consensus regarding the definition of older adults and how to incorporate age as a de-escalation factor.

Although some studies have shown that the risk of a local recurrence is lower in older patients, and the benefits of RT following breast-conserving surgery decline with age [27,28], most clinicians in this survey tended to offer RT (80%). It is important to note that the small number of clinicians answering this question may limit our ability to extrapolate. However, some will take into account the life expectancy of the patient and whether or not they will receive ET in their decision-making. Indeed, Cancer and Leukemia Group B [CALGB] 9343 and PRIME-II have suggested that the omission of RT in this subset is an acceptable strategy, assuming that ET is administered [5,6]

According to guidelines, adjuvant ET should be offered to all patients with HR+ breast tumors > 0.5 cm, regardless of age, provided they are candidates for medical therapy [1]. In this study, most respondents would offer ET (77%), and the majority of them would choose an AI. This is supported by the Early Breast Cancer Trialists’ Group (EBCTCG) meta-analysis which showed that AIs are slightly superior for reducing relapse risk, compared with tamoxifen, even in patients aged 70 years or older [29]. Although some patients might be over-treated with ET, there is clearly a lack of data to suggest omitting ET. Indeed, older patients may have other health issues that impact their survival more than breast cancer, and therefore the benefit of ET is diminished [14].

In the last part of the survey, respondents were presented with a series of questions related to the design of a future clinical trials geared toward studying the clinical benefits of de-escalated adjuvant ET and/or RT, and the relevance of various potential study endpoints to facilitate the design of practice-changing trials. The results strongly suggest that the physicians surveyed were very interested in such trials. As most of the previous de-escalation trials studied the omission of RT, it is striking that a future trial comparing adjuvant ET vs. RT plus ET is deemed to be the most informative and impactful design for the majority of respondents. DFS and OS were the preferred primary study endpoints and quality of life was the most important secondary endpoint. Another de-escalation strategy for adjuvant therapy in older patients with low-risk breast cancer is to shorten the duration of ET. This was not questioned in this survey, but there is an ongoing phase 2 trial, comparing 5 versus 2 years of ET in low-risk patients aged > 50 (NCT03917082). There is a second ongoing Canadian trial on this topic, the REaCT-70 study, that is evaluating the harms and benefits of endocrine therapy in older patients with lower-risk breast cancer (NCT04921137).

The study received fewer responses than desired, but this is consistent with the response rates observed in many surveys. This antipathy to surveys is likely more pronounced due to the COVID-19 pandemic. As with all surveys, there is an inherent selection bias in those that are contacted and in those that respond. The physicians that completed this survey might be more involved in research and inclined to participate in studies. Therefore, the number of physicians that are willing to take part in de-escalation studies might be overestimated.

## 5. Conclusions

The results from this study highlight the clinical importance of evaluating the harms and benefits the adjuvant therapies in patients ≥ 70 years of age with low-risk HR+/HER2− EBC. It will serve as a useful guide in the design of future clinical trials and increase the likelihood that trial results will have a clinical impact. Given that there is no robust data supporting the optimal management (adjuvant RT alone vs. adjuvant ET alone vs. adjuvant RT plus ET) of older patients with low-risk HR+/HER2− EBC, this feedback from Canadian oncologists will be of great value [30].

## Figures and Tables

**Table 1:** Physician demographics.

Demographics	N	N (%)
Profession:	49	
Medical Oncologist		30 (60)
Radiation Oncologist		12 (24)
Surgical Oncologist		4 (8)
General Practitioner in Oncology		1 (2)
Nurse Practitioner in Oncology		1 (2)
General internist doing medical oncology		1 (2)
Time in independent practice:	50	
5 years or less		14 (28)
6–10 years		9 (18)
11–20 years		12 (24)
More than 20 years		15 (30)
Work Setting:	50	
An academic (teaching) hospital with a cancer center		38 (76)
A non-academic (community) hospital with a cancer center		8 (16)
An academic hospital without cancer center		4 (8)

**Table 2:** Perception regarding definition of “low risk” ER+/HER2− EBC and “older”.

Key Definition Items	N	N (%)
Participants opinion regarding the definition of lower-clinical-risk disease, proposed by Sparano et al. ^1^	50	
Agree		34 (68)
Don’t agree		5 (10)
Unsure		11 (22)
Opinion regarding the exclusion of multifocal breast cancer in the low-risk definition	49	
Yes		9 (18)
No		36 (73)
Unsure		4 (8)
Opinion on requiring Ki-67 to define low risk	50	
Yes		3 (6)
Maybe		28 (56)
No		16 (36)
Unsure		3 (6)
Opinion on requiring multigene profiling assays to define low risk	50	
Yes		10 (20)
Maybe		25 (50)
No		13 (26)
Unsure		2 (4)
Preferred multigene profiling to inform low-risk definition	35	
Oncotype Dx Rs		26 (74)
Prosigna		0
Endopredict		0
Any of the 3 aforementioned		9 (26)
No		0
Other		0
Oncotype Dx Recurrence score cut-off value to define low risk	35	
<11		4 (11)
<16		3 (9)
<18		9 (26)
<26		11 (31)
Depends on age		6 (17)
Unsure		2 (6)
Chronological age to define older	49	
65		1 (2)
70		22 (45)
75		10 (20)
80		15 (31)
85		1 (2)
Biological age to define older	47	
65		1 (2)
70		21 (45)
75		7 (15)
80		14 (30)
85		4 (8)

^1^ Defined node-negative low-risk breast cancer as: a tumor ≤ 3 cm with a low histological grade, a tumor ≤ 2 cm with an intermediate grade, or a tumor ≤ 1 cm with a high grade.

**Table 3:** Physician personal practice and perception with respect to adjuvant RT and ET use in patients aged 70 and older with low-risk HR+/HER2− EBC.

Practice Patterns and Perceptions	N	N (%)
Recommendations of RT for patients 70 years or older with low-risk, node-negative ER−positive/HER2−negative breast cancer	12	
• Radiation is strongly recommended, REGARDLESS of whether patient receives ET or not		1 (8)
• Radiation is strongly recommended if the patient is NOT getting ET		1 (8)
• Radiation is generally recommended REGARDLESS of whether patient receives ET or not		1 (8)
• Radiation is generally recommended if the patient is NOT getting ET		2 (17)
• Radiation is offered only if the patient has a reasonable life expectancy and few co-morbidities, REGARDLESS of whether patient receives ET or not		4 (33)
• Radiation is offered only if the patient has a reasonable life expectancy and few co-morbidities, and if the patient is NOT getting ET		1 (8)
• Radiation is not recommended for this population regardless of life expectancy or co-morbidities		0
• Others		2 (17)
RT regimen recommended for patients 70 years or older with low risk, node-negative ER−positive/HER2−negative breast cancer	12	
• 5 days per week for 3 weeks (+/− boost)		3 (25)
• 5 days per week for 5 weeks (+/− boost)		0
• Weekly for 5 weeks		0
• Biweekly for 2.5 weeks		0
• Accelerated partial breast radiation (external beam or brachytherapy)		4 (33)
• Standard fractionation partial breast (ex: IMPORT LOW)		1 (8)
• Other		4 (33)
RT tolerability compared with younger patients	12	
• Worse		3 (25)
• Not worse/Same		9 (75)
• Unsure		0
Adherence of older patient to RT	12	
• Never		2 (17)
• <1% of patients		4 (33)
• 1 to <5%		5 (42)
• 5 to 10%		0
• 10% of patients		1 (8)
• Others		0
Recommendations of ET for patients 70 years or older with low risk, node-negative ER−positive/HER2−negative breast cancer	44	
• ET is strongly recommended		9 (20)
• ET is generally recommended		25 (57)
• ET is not recommended if the patient has multiple co-morbidities		7 (16)
• ET is not recommended for this population regardless of performance status or co-morbidities		0
• Other: discuss risk and benefit with the patient		3 (7)
Type of ET	45	
• Aromatase inhibitor, in the majority of cases		22 (49)
• Tamoxifen, in the majority of cases		6 (13)
• Switch strategy (tamoxifen and aromatase inhibitor), in the majority of cases		14 (31)
• Other		3 (7)
Duration of ET	44	
• 5 years		15 (34)
• 5 years but with low threshold to stop therapy if side effects occur		26 (59)
• 7 years		1 (2)
• 10 years		0
• No ET recommended		0
• Others: 5 to 7 years, discuss with the patient		2 (5)
Adherence of older patients to ET:	46	
• More 90% of patients are fully adherent to their ET		5 (11)
• More than 75% of patients are fully adherent to their ET		18 (39)
• More than 50% of patients are fully adherent to their ET		19 (41)
• Unsure		4 (9)

**Table 4:** Views on potential de-escalation studies.

De-Escalation Study Views and Features	N	N (%)
Are trials to evaluate the risks and benefits of RT and ET in older patients with low-risk, node-negative ER−positive/HER2−negative breast cancer needed?	49	
Yes		38 (78)
No		6 (12)
Unsure		5 (10)
The most informative and impactful trial design based on participants knowledge, experience and current practice:	38	
• Adjuvant RT alone VERSUS Adjuvant ET alone		15 (39)
•Adjuvant ET alone VERSUS Adjuvant RT plus ET		11 (29)
• Adjuvant RT alone VERSUS Adjuvant RT plus ET		12 (32)
Choice of first most important study endpoint (one only):	48	
• Ipsilateral breast recurrence		3 (6.5)
• Locoregional recurrence		4 (8)
• Distant recurrence-free survival		7 (15)
• Disease-free survival		12 (25)
• Breast cancer specific survival		7 (15)
• Overall survival		9 (19)
• Time to treatment failure		2 (4)
• Indicator of health quality of life using EORTC-QLQ-C30 questionnaire		3 (7)
• Other		1 (2)
Second most important clinical endpoints (more than one):	48	
• Indicator of specific adverse events using CTCAE v5 1		18 (37)
• Indicator of specific adverse events using PRO-CTCAE 2		16 (33)
• Time from randomization to discontinuation of therapy from any causes		15 (31)
• Indicator of health quality of life using EORTC-QLQ-C30 questionnaire		26 (54)
• Ipsilateral breast recurrence		6 (12)
• Locoregional recurrence		15 (31)
• Distant recurrence-free survival		15 (31)
• Disease-free survival		19 (39)
• Breast cancer specific survival		16 (33)
• Overall survival		20 (41)
• Time to treatment failure		12 (25)

^1^ Common Terminology Criteria for Adverse Events version 5; ^2^ Patient Report Outcome—Common Terminology Criteria for Adverse Events.

## Data Availability

Data is available from the author upon request and with permission from the Ontario Cancer Research Ethics Board.

## Supplementary Materials

The following are available online at https://www.mdpi.com/article/10.3390/curroncol29010001/s1: File S1: Study protocol and Physician’s survey.
