# Supplementary material for: Management Strategies for Older Patients with Low-Risk Early-Stage Breast Cancer: A Physician Survey
Source: Curr Oncol. 2021 Dec 21;29(1):1–13. doi: 10.3390/curroncol29010001 (PMC8774930; doi:10.3390/curroncol29010001)
Supplement: Supplementary file 1 [file curroncol-29-00001-s001.zip › curroncol-1465082-supplementary.pdf]

Oct 20, 2020

SUPPLEMENTARY MATERIALS

Study Protocol

First, do no harm. Evaluating the harms and benefits of radiotherapy and endocrine therapy in patients ≥70 years of age with low risk breast cancer. (REaCT-70 Survey)

STUDY PROTOCOL

Primary investigators: Dr. Marie-France Savard, Dr. Lynn Chang and Dr. Angel Arnaout

BACKGROUND:

Current guidelines recommend adjuvant radiotherapy and endocrine therapies for estrogen receptor (ER)-positive early stage breast cancer[1]. In a subset of the patients, the risks and side effects of taking radiotherapy and/or endocrine therapies may outweigh the benefits. This is a particular concern for elderly patients with a low risk breast cancer and comorbid medical conditions[2].

Clinical factors such as low-intermediate grade, tumor size ◎2 cm, and older age has been used successfully in a few studies to identify patients with a low risk of recurrence[3–7]. For instance, in the CALGB 9343 trial, despite the omission of adjuvant radiotherapy, the local and regional recurrence ratesremains below 5% at 5 years and below 10% at 10 years without significant differences in time to mastectomy or , distant metastasis or breast cancer specific survival, in women ≥70 years with T1N0 breast cancer treated with adjuvant tamoxifen[4]. Furthermore, some studies suggest the existence of a less aggressive breast cancer biology in most patients ≥70 years of age[8].

Even though 30% of early stage breast cancers are diagnosed in patients ≥70 years , the elderly are oftenunderrepresented in large prospective clinical trials evaluating the benefits of adjuvant therapies[2,9]. Acknowledging this underrepresented group, the FDA recently published a guidance for industry, named*Inclusions of Older Adults in Cancer Clinical Trials,* to assist and encourage stakeholders in the adequate representation of older adults in cancer clinical trials[10].

The acceptable benefit-risk profile of adjuvant radiotherapy and endocrine

therapy remains to be definedfor elderly patients with low risk breast cancer. We will conduct a survey among oncologists (i.e. medical oncologists, radiation oncologists, surgical oncologists and general practitioners in oncology/radiation) and older patients with a low risk breast cancer to address this issue and to determine the best adjuvant treatment de-escalation strategies. The information obtained from these surveys will help us to develop apragmatic clinical trial that evaluates the risks and benefits of adjuvant radiotherapy and endocrine therapies in older patients with low risk breast cancer. The answers of the respondents will help us to alignour research objectives with their priorities and integrate their voice into our research projects. We hope to use these responses to make future clinical studies more clinically relevant, patient-centered and practice-changing.

OBJECTIVES:

To evaluate physician and patient perceptions regarding:

1) The current practices involving adjuvant endocrine and radiotherapy in elderly patients with an ER-positive low risk breast cancer;
2) The side effects and impact on quality of life of adjuvant radiotherapy and endocrine therapy in elderlypatients with an ER-positive low risk breast cancer;
3) The most appropriate de-escalating design and endpoints for a study evaluating risk and benefits ofradiotherapy and endocrine therapy in elderly patients with an ER-positive low risk breast cancer
4) The importance of a future study evaluating risk and benefits of radiotherapy and endocrine therapyin elderly patients with an ER-positive low risk breast cancer.

METHODS:

Patient Survey

The survey will be explained to prospective participants during their clinic visit by a member of their circle of care (e.g. medical oncologists, radiation oncologists, surgical oncologists, general practitioners in oncology/radiation and nursing staff) and if interested, the patient will be given a paper copy of the survey and the information sheet. If the patient wishes for the survey to be emailed to them (and if permitted by the institution), then the study research assistant will send it from a secure hospital server. Patients will receive an email with the link to the survey on Microsoft Forms (on the Ottawa Hospital SharePoint site) which can be completed online. Or they can request to receive

the questionnaire by emailas a Word file or PDF file and can highlight their answers and email the completed survey back to the study staff. Surveys sent by email will be printed in hard copy (with no identifiers) and filed with the other paper copies. The survey can be completed at any time the patient wishes. The survey is anonymous and does not collect any personal identifying information. The questionnaire is anticipated to take 5-10 minutes to complete. Patients from three sites in Ontario will be participating.

Inclusion criteria

1) Patients with an ER-positive early stage (node negative) breast cancer treated with a lumpectomy ormastectomy;
2) Patients who were offered radiotherapy AND endocrine therapy, such as tamoxifen, anastrozole(*Arimidex*), letrozole (*Femara*) or exemestane (*Aromasin*);
3) Patients who are ≥70 years of age;
4) Able to provide verbal consent;
5) Willing and able to complete a survey in English.

Physician Survey

Physicians involved in the treatment of patients with ER-positive early stage (node-negative) breast cancer(e.g. medical oncologists, radiation oncologists, surgical oncologists and general practitioners in oncology/radiation) will be approached to participate in the physician survey. The Ottawa site has a collection of publicly available physicians email addresses that have been used in previous surveys of thistype. The online survey will be run using Microsoft Forms on the Ottawa Hospital SharePoint site. Physicians will be emailed an information sheet and a link to the survey designed by the study team, whichis anticipated to take 5-10 minutes to complete. The survey is completed anonymously (no personal identifiers will be collected).

Inclusion criteria

1) Physicians involved in the adjuvant treatment with radiotherapy and/or endocrine therapy of patientswith an ER-positive early stage (node-negative) breast cancer, such as medical oncologists, radiation oncologists, surgical oncologists and general practitioners in oncology/radiation.
2) Willing and able to complete a survey in English.

STUDY ENDPOINTS:

Indicators physician and patient perceptions regarding:

1) The current practices involving adjuvant endocrine and radiotherapy in elderly patients with an ER-positive low risk breast cancer;
2) The side effects and impact on quality of life of adjuvant radiotherapy and endocrine therapy in elderlypatients with an ER-positive low risk breast cancer;
3) The most appropriate de-escalating design and endpoints for a study evaluating risk and benefits ofradiotherapy and endocrine therapy in elderly patients with an ER-positive low risk breast cancer;
4) The importance of a future study evaluating risk and benefits of radiotherapy and endocrine therapyin elderly patients with an ER-positive low risk breast cancer.

TIMELINE:

- *REB Submission*: April 2020
- *Data Collection: June-August 2020*
- *Statistical Analysis: September 2020*
- *Dissemination of Results: Fall 2020*

RECRUITMENT:

These surveys are expected to recruit 200 patients and 50 physicians. Participants will be asked to complete the survey in English.

DATA COLLECTION AND ANALYSIS:

Data collected in this study will include paper copies of the survey completed by patients, which will be secured in a locked filing cabinet at each local institution. Physicians willing to participate will be provided with the opportunity to complete the questionnaire online. All responses will be anonymous, and data willbe stored with a platform that provides security, with password-limited access provided to the study teamonly. Collected data will be used to generate a database that will be managed using spreadsheet software (e.g. Excel) and digitally saved in a TOH encrypted server. Data analysis will generate current practice patterns in Ontario and also obtain the views of physicians and patients on acceptable benefit-risk profilesfor adjuvant radiation and endocrine therapies as well as the best adjuvant treatment de-escalation strategyfor elderly patients with low risk breast cancer. It will be presented descriptively.

FUNDING:
All costs associated with this study will be covered by internal funds, with no industry or pharmaceuticalfunding involved.

REFERENCES:

1. National Comprehensive Cancer Network. NCCN Clinical Practice Guidelines in Oncology:Breast Cancer, Version 3.2020. (2020). Available at: https://www.nccn.org/professionals/physician_gls/pdf/breast.pdf.
2. Lee, S. & Seo, J. H. Current Strategies of Endocrine Therapy in Elderly Patients with BreastCancer. *Biomed Res. Int.* **2018**, 1–12 (2018).
3. Fisher, B. *et al.* Tamoxifen, Radiation Therapy, or Both for Prevention of Ipsilateral BreastTumor Recurrence After Lumpectomy in Women With Invasive Breast Cancers of One Centimeter or Less. *J. Clin. Oncol.* **20**, 4141–4149 (2002).
4. Hughes, K. S. *et al.* Lumpectomy Plus Tamoxifen With or Without Irradiation in Women Age 70Years or Older With Early Breast Cancer: Long-Term Follow-Up of CALGB 9343. *J. Clin. Oncol.* **31**, 2382–2387 (2013).
5. Sparano, J. A. *et al.* Clinical and Genomic Risk to Guide the Use of Adjuvant Therapy for BreastCancer. *N. Engl. J. Med.* **380**, 2395–2405 (2019).
6. Kunkler, I. H., Williams, L. J., Jack, W. J. L., Cameron, D. A. & Dixon, J. M. Breast-conservingsurgery with or without irradiation in women aged 65 years or older with early breast cancer (PRIME II): a randomised controlled trial. *Lancet Oncol.* **16**, 266–273 (2015).
7. Fyles, A. W. *et al.* Tamoxifen with or without Breast Irradiation in Women 50 Years of Age orOlder with Early Breast Cancer. *N. Engl. J. Med.* **351**, 963–970 (2004).
8. Downs-Canner, S. M. *et al.* Nodal positivity decreases with age in women with early-stage, hormone receptor–positive breast cancer. *Cancer* **126**, 1193–1201 (2020).
9. DeSantis, C. E. *et al.* Breast cancer statistics, 2015: Convergence of incidence rates betweenblack and white women. *CA. Cancer J. Clin.* **66**, 31–42 (2016).
10. FDA. Inclusion of Older Adults in Cancer Clinical Trials Draft Guidance for Industry. *FDA-2019-D-5572* (2020). Available at: https://www.fda.gov/regulatory-information/search-fda- guidance-

documents/inclusion-older-adults-cancer-clinical-trials.

Physician's Survey

Survey Title: First, do no harm. Evaluating the harms and benefits of radiotherapy and endocrine therapy in patients ≥70 years of age with low risk breast cancer. (REaCT-70 Survey)

A Survey for Physicians

In a subset of patients with estrogen receptor-positive/HER2-negative breast cancer, the risks and side effects of taking endocrine therapy may outweigh the benefits. This is particularly a concern for elderly patients with low risk breast cancer for whom medical comorbidities may be significant. Even though 30% of early stage breast cancers are diagnosed in patients ≥70 years of age, elderly patients are underrepresented in large prospective clinical trials evaluating adjuvant endocrine therapy. Furthermore, some studies suggested the existence of a less aggressive biology in breast cancer in patients ≥70 years of age. Therefore, the overall benefit of adjuvant endocrine therapy remains to be determined for this population.

We invite you to participate in this survey to determine the common practises and perceptions regarding the use of endocrine therapy in elderly patients with a low risk breast cancer. Based on your expert input, we hope to incorporate your responses into making future clinical studies that are more clinically relevant and practice changing.

The study is independently funded and anonymous, with no links to either you or your institution, and all results will be presented in aggregate. Completion of the survey implies your consent to participate in this survey.

This survey should take no more than 10-20 minutes of your time.

We thank you for your support and are confident that your answers will assist us in developing more standard treatment policies for Canadian patients.

Oct 20, 2020

Assessment of survey eligibility

1. Do you discuss/prescribe endocrine and/or radiation therapy for patients with breast cancer?
   a) Yes
   b) No

<u>IF YOU ANSWERED NO TO THIS QUESTION YOU CAN END THE SURVEY HERE</u>.

Demographics

2. Please identify your profession:
   a) Medical Oncologist
   b) Radiation Oncologist
   c) Surgical Oncologist that initiates endocrine therapy
   d) Surgical Oncologist that does not initiate endocrine therapy
   e) General Practitioner in Oncology/Radiation
   f) Other; please specify:
   ____

3. In what setting do you predominantly work?
   a) An academic (teaching) hospital with a cancer centre
   b) A non-academic (community) hospital with a cancer centre
   c) An academic hospital without a cancer centre
   d) A non-academic hospital without a cancer centre
   e) Private practice
   f) Other; please specify:
   ____

4. How many years have you been practicing in your field post residency / fellowship?
   a) ≤ 5 years
   b) 6-10 years
   c) 11-20 years
   d) > 20 years

Institutional practices regarding initiation of endocrine therapy

5. In your institution, who is typically responsible for initiating adjuvant endocrine therapy?

a) Medical oncologist
b) Radiation oncologist
c) Surgical oncologist
d) General Practitioner in Oncology (GPO)
e) Varies
f) Other; please specify:____

Defining low risk disease in patients with node-negative ER-positive/HER2-negative breast cancer

6. Based on the definition of low clinical risk proposed by Sparano et al. in the article Clinical and Genomic Risk to Guide the Use of Adjuvant Therapy for Breast Cancer (NEJM 380 (25). June 2019), do you agree with defining node-negative ER-positive /HER2-negative low risk breast cancer as:

- Tumor ≤3cm with a low histological grade, or
- Tumor ≤2cm with intermediate grade, or
- Tumor ≤1cm with high grade.

a) Yes
b) No
c) Unsure

7. If you answered NO or UNSURE, please mark in the table below what you feel are the appropriate definitions of low risk, node negative ER-positive/HER2-negative breast cancer:

Size of tumor	Low histologic grade	Intermediate histologic grade	High histologic grade
≤1 cm			
≤2 cm			
≤3 cm			
≤5 cm			

8. Would you exclude multifocal breast cancer in the low risk breast cancer definition?
   a) Yes, multifocal breast cancer should be excluded from the definition of low risk breast cancer
   b) No, some multifocal breast cancers can be considered as low risk breast cancer
   c) Unsure

9. Irrespective of tumour size and grade, would you require a Ki-67 result to define low risk breast cancer?
   a) Yes
   b) Maybe, Ki-67 will improve my confidence in identifying low risk breast cancer but is not absolutely necessary.
   c) No, I don't think a Ki-67 is necessary for identifying low risk breast cancer
   d) Unsure

10. Irrespective of tumour size and grade, would you require one of the multigene profiling assays (MPA) such as Oncotype Dx Recurrence Score (RS), EndoPredict and Prosigna to define low risk breast cancer?
   a) Yes, I would not feel comfortable defining a node-negative ER-positive /HER2-negative breast cancer as low risk without a MPA
   b) Maybe, the MPA will improve my confidence in identifying low risk breast cancer but is not absolutely necessary.
   c) No, I don't think an MPA is necessary for identifying low risk breast cancer
   d) Unsure

1. If you answered a) or b) to Question 10, which MPAs would you prefer?
   a) Oncotype Dx RS
   b) Prosigna
   c) EndoPredict
   d) Any of Oncotype Dx RS, Prosigna or Endopredict
   e) I do not use these tests
   f) Other, please specify:____

12. If you answered a) or d) to Question 11, which Oncotype Dx Recurrence Score cut-off would you consider in the definition of low risk breast cancer?
   a) <11
   b) <16
   c) <18
   d) <26

e) ≤30
f) Unsure
g) Other, please specify:______________________________

13. If you answered b) or d) to Question 11, which Prosigna cut-off would you consider in the definition of low risk breast cancer?
a) <41
b) <61
c) Unsure
d) I do not use this test
e) Other, please specify:______________________________

14. If you answered c) or d) to the Question 11, which EPclin Risk Score cut-off would you consider in the definition of low risk breast cancer?
a) < 3.3287
b) Unsure
c) I do not use this test
d) Other, please specify:______________________________

Defining the term "elderly"

As you know, patients can have both a chronological age (i.e. the number of years a person has been alive) and a biological age also referred to as physiological age (i.e. taking lifestyle factors and comorbidities into account).

15. What chronological age would you define as "elderly"?
a) ≥_______ years old

16. Do you have any comments to add to the question above? ____

17. What biological age would you define as "elderly"?
a) ≥_______ years old

18. Do you have any comments to add to the question above? ____

19. At what age would you consider de-escalating (eg partial breast radiation, altered fractionation) and/or omitting (eg not offering) radiotherapy based on age? ≥_______ years old

20. Is this chronological age or biological age?
    a) chronological
    b) biological age

21. Do you have any comments to add on the above 2 questions? ______________________________

22. At what age would you consider de-escalating (eg a shorter duration of therapy) and/or omitting (eg not offering) endocrine therapy based on age? ≥_______ years old

23. Is this chronological age or biological age?
    a) chronological
    b) biological age

24. Do you have any comments to add on the above 2 questions? ______________________________

IF YOU ARE A RADIATION ONCOLOGIST, PLEASE ANSWER THE QUESTIONS 25 TO 30. IF NOT, PLEASE GO TO QUESTION 31.

QUESTIONS FOR PHYSICIANS THAT INITIATE RADIOTHERAPY

The following questions relate to your personal practice regarding the use of radiotherapy in node-negative ER-positive/HER2-negative low risk breast cancer in elderly patients. In the next questions for consistency we are using elderly as those patients with a chronological age of ≥70 years.

25. In patients ≥70 years with low risk, node-negative ER-positive/HER2-negative breast cancer, do you recommend radiotherapy?
    a) I strongly recommend radiotherapy, REGARDLESS of whether patient receives endocrine therapy or not
    b) I strongly recommend radiotherapy, if the patient is NOT getting endocrine therapy

c) I generally recommend radiotherapy, REGARDLESS of whether patient receives endocrine therapy or not
d) I generally recommend radiotherapy, if the patient is NOT getting endocrine therapy
e) I offer radiotherapy only if the patient has a reasonable life expectancy and few co-morbidities, REGARDLESS of whether patient receives endocrine therapy or not
f) I offer radiotherapy only if the patient has a reasonable life expectancy and few co-morbidities, and if the patient is NOT getting endocrine therapy
g) I do not recommend radiotherapy for this population regardless of life expectancy or co-morbidities
h) Other, please specify:____

26. In patients ≥70 years with low risk, node-negative ER-positive/HER2-negative breast cancer, what radiotherapy regimen do you most commonly offer?
a) 5 days per week for 3 weeks (+/- boost)
b) 5 days per week for 5 weeks (+/- boost)
c) Weekly for 5 weeks
d) Biweekly for 2.5 weeks
e) Accelerated partial breast radiation (external beam or brachytherapy)
f) Standard fractionation partial breast (ex: IMPORT LOW)
g) Other, please specify: ________________________________

27. In patients ≥70 years with low risk, node-negative ER-positive/HER2-negative breast cancer, do you offer a radiation boost in addition to one of the above regimens?
a) Yes, always
b) No, never
c) Depends on pathology (ex: margins, grade, LVI)

28. Some surgeons follow the Society of Surgical Oncology Guidelines in not offering sentinel node biopsy or any form of axillary surgery to patients ≥70 years. How would this affect your decision to give radiotherapy?
a) More likely to give radiotherapy
b) Less likely to give radiotherapy
c) Does not affect my decision

29. How often do you encounter problems with radiotherapy compliance amongst patients ≥70 years?
   a) Never
   b) < 1% of patients
   c) 1 to <5% of patients
   d) 5 to 10%
   e) 10% of patients
   f) Other – please comment ____________________________________

30. In patients ≥70 years, do you believe radiotherapy tolerability is worse than in younger patients?
   a) Yes
   b) No
   c) Same
   d) Unsure

<u>IF YOU PRESCRIBE ENDOCRINE THERAPY TO PATIENTS WITH BREAST CANCER PLEASE ANSWER THE QUESTIONS 31 TO 39.</u>
<u>IF NOT, PLEASE GO TO QUESTION 40.</u>

<u>QUESTIONS FOR PHYSICIANS THAT INITIATE ENDOCRINE THERAPY</u>
Personal practice regarding the use of endocrine therapy in node-negative ER-positive/HER2-negative low risk breast cancer in elderly patients

31. In patients ≥70 years of age with low risk, node-negative ER-positive/HER2-negative breast cancer, do you recommend endocrine therapy?
   a) I strongly recommend endocrine therapy
   b) I generally recommend endocrine therapy
   c) I do not offer endocrine therapy if the patient has multiple co-morbidities
   d) I do not recommend endocrine therapy for this population regardless of performance status or co-morbidities
   e) Other, please specify:____
   _

32. In patients ≥70 years with low risk, node-negative ER-positive/HER2-negative breast cancer, what duration do you recommend for endocrine therapy?
   a) 5 years
   b) 5 years, but with a low threshold to stop therapy if side effects occur
   c) 7 years

d) 10 years
e) I don't recommend endocrine therapy for this population
f) Other, please specify:______________________________________

33. In patients ≥70 years with low risk node-negative ER-positive/HER2-negative breast cancer, who have no contraindications to any treatment, which is your preferred choice of endocrine therapy?
a) An aromatase inhibitor, in the majority of cases
b) Tamoxifen, in the majority of cases
c) Switch strategy (Tamoxifen and an AI), in the majority of cases
d) Other: _________________________________

34. Some surgeons follow the Society of Surgical Oncology Guidelines in not offering sentinel node biopsy or any form of axillary surgery to patients ≥70. How would this affect your decision to give endocrine therapy?
a) More likely to give endocrine therapy
b) Less likely to give endocrine therapy
c) Does not affect my decision

35. If you have recommended endocrine therapy, how often do you think that patients ≥70 years do not actually ever start endocrine therapy?
a) They always start their endocrine therapy
b) < 10% of patients never start their endocrine therapy
c) < 25% of patients never start their endocrine therapy
d) < 50% of patients never start their endocrine therapy
e) Unsure
f) Other – please state: _______________________________________

36. How adherent or compliant are patients ≥70 years to endocrine therapy (i.e. taking medication as prescribed, remembering to take **medication** on time, and understanding the directions)?
a) They always take all their endocrine therapy as prescribed
b) ≥90% of patients are fully adherent to their endocrine therapy
c) ≥75% of patients are fully adherent to their endocrine therapy
d) ≥ 50% of patients are fully adherent to their endocrine therapy
e) Unsure
f) Other – please state: ____

37. How often do you think patients ≥70 years stop taking their endocrine therapy earlier than the originally prescribed duration?

a) They always take their full duration of endocrine therapy
b) < 10% of patients will stop endocrine therapy early
c) < 25% of patients will stop endocrine therapy early
d) < 50% of patients will stop endocrine therapy early
e) Unsure
f) Other – please state: ____

38. In patients ≥70 years, do you believe that endocrine therapy tolerability is worse than in younger patients?
a) Yes
b) No
c) Unsure

39. Which of the following issues interfere the most with adjuvant endocrine therapy compliance rates in patients ≥70 years with a low risk breast cancer? Please rank your top 3 most important issues with 1 being the most important.

Patient considers the benefit of endocrine therapy to be minimal	
Immediate side effects	
Long term side effects	
Long duration of treatment	
Patient does not have support from family or friends	
Other co-morbidities (eg dementia) that prevent them from taking medication	
Polypharmacy	
Patient does not understand their disease or the therapy	
Patient does not want to take medication	
Other	

Considerations for a potential clinical trial in elderly patients with node-negative ER-positive/HER2-negative low risk breast cancer

40. Are trials to evaluate the risks and benefits of radiotherapy and endocrine therapy in elderly patients with low risk, node-negative ER-positive/HER2-negative breast cancer needed?
a) Yes
b) No
c) Unsure

41. If no or unsure, please comment: ____

42. If yes, based on YOUR knowledge, YOUR experience and YOUR current practice, which trial design would be the most informative and impactful?

Trials' Arms	
Adjuvant radiotherapy alone VERSUS Adjuvant endocrine therapy alone	
Adjuvant endocrine therapy alone VERSUS Adjuvant radiotherapy plus endocrine therapy	
Adjuvant radiotherapy alone VERSUS Adjuvant radiotherapy plus endocrine therapy	

43. Would you feel comfortable offering one of the following trials that randomizes elderly patients with low risk node-negative ER-positive/HER2-negative breast cancer?

Trials' Arms	Yes	No	Unsure
Adjuvant radiotherapy alone<br>VERSUS<br>Adjuvant endocrine therapy alone			
Adjuvant endocrine therapy alone<br>VERSUS<br>Adjuvant radiotherapy plus endocrine therapy			
Adjuvant radiotherapy alone<br>VERSUS<br>Adjuvant radiotherapy plus endocrine therapy			

44. If no or unsure, please comment: ____

45. Which primary endpoint do you think is the **most** appropriate in a study evaluating risks and benefits of endocrine therapy in elderly patients with node-negative ER-positive/HER2-negative low risk breast cancer? Choose only **ONE**

a) Ipsilateral breast recurrence (defined as time from randomization until time disease recurrence in the ipsilateral breast)
b) Locoregional recurrence (defined as time from randomization until time disease recurrence in the ipsilateral breast, axillary node, infraclavicular node or supraclavicular node)

c) Distant recurrence-free survival (defined as time from randomization until time disease recurrence developing beyond the ipsilateral or contralateral breast, chest wall, or regional lymph node including ipsilateral axillary, supraclavicular, or internal mammary lymph node)
d) Disease-free survival (defined as time from randomization until time to any disease recurrence or death from any cause)
e) Breast cancer specific survival (defined as time from randomization until locoregional or distant breast cancer recurrence)
f) Overall survival (defined as the time from randomization until death from any cause)
g) Time to treatment failure (defined as time from randomization to discontinuation of treatment for any reason, including disease progression, treatment toxicity, and death)
h) Indicator of health quality of life using EORTC-QLQ-C30 questionnaire
i) Other, please specify:____

46. Which secondary endpoint is the most appropriate to include in a study evaluating risks and benefits of endocrine therapy in elderly patients with node-negative ER-positive/HER2-negative low risk breast cancer?

a) Indicator of specific adverse events using CTCAE v5 (Common Terminology Criteria for Adverse Events version 5)
b) Indicator of specific adverse events using PRO-CTCAE (Patient Report Outcome Common Terminology Criteria for Adverse Events)
c) Time from randomization to discontinuation of therapy from any causes
d) Indicator of health quality of life using EORTC-QLQ-C30 questionnaire
e) Ipsilateral breast recurrence (defined as time from randomization until time disease recurrence in the ipsilateral breast)
f) Locoregional recurrence (defined as time from randomization until time disease recurrence in the ipsilateral breast, axillary node, infraclavicular node or supraclavicular node)
g) Distant recurrence-free survival (defined as time from randomization until time disease recurrence developing beyond the ipsilateral or contralateral breast, chest wall, or regional lymph node including ipsilateral axillary, supraclavicular, or internal mammary lymph node)
h) Disease-free survival (defined as time from randomization until time to any disease recurrence or death from any cause)
i) Breast cancer specific survival (defined as time from randomization until locoregional or distant breast cancer recurrence)

j) Overall survival (defined as the time from randomization until death from any cause)
k) Time to treatment failure (defined as time from randomization to discontinuation of treatment for any reason, including disease progression, treatment toxicity, and death)
l) Other, please specify:____

Thank you for participating in this survey!
